# Design of Porous 3D Interdigitated Current Collectors and Hybrid Microcathodes for Zn-Ion Microcapacitors

**DOI:** 10.1021/acsnano.5c00917

**Published:** 2025-03-25

**Authors:** Yujia Fan, Nibagani Naresh, Yijia Zhu, Mingqing Wang, Buddha Deka Boruah

**Affiliations:** Institute for Materials Discovery, University College London (UCL), London WC1E 7JE, U.K.

**Keywords:** Zn-ion capacitors, porous interdigitated electrodes, hybrid material loading, fast ion diffusion, synergistic enhancements

## Abstract

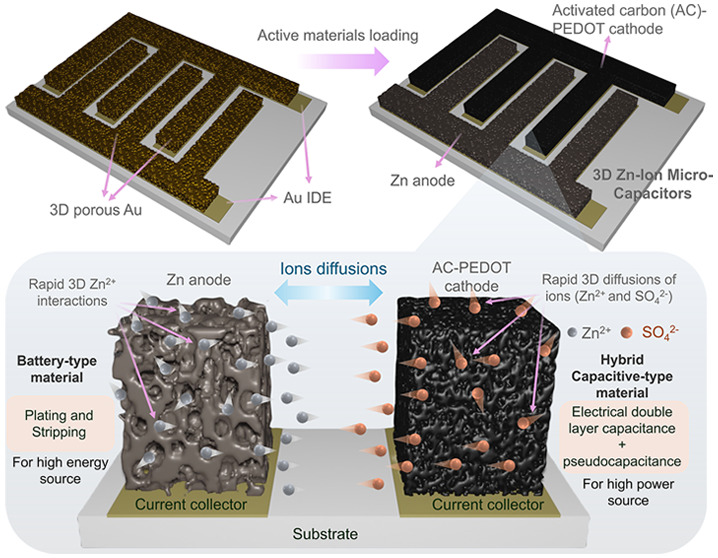

Zinc-ion microcapacitors (ZIMCs) have gained considerable attention for their intrinsic charge storage mechanisms, combining a battery-type anode with a capacitor-type cathode. However, their development is constrained by challenges related to electrode material selection and microscale device design, especially given the limited footprint of such devices. Despite their potential, exploration of smart electrode processing and hybrid materials for on-chip ZIMCs remains limited. In this work, we introduce 3D gold interdigitated electrodes (3D Au IDEs) as highly porous current collectors, loaded with zinc (Zn) as the anode and hybrid activated carbon coated with PEDOT (AC-PEDOT) as the cathode, using an advanced microplotter fabrication technique. Compared with planar Zn//AC ZIMCs, where Zn and AC materials are loaded onto planar Au IDEs, the 3D Au Zn//AC-PEDOT ZIMCs demonstrate significantly enhanced performance. This is attributed to the critical role of IDEs in increasing the charge storage capacity, improving long-term cycling stability, and boosting capacitive-controlled charge storage contributions. The 3D Au Zn//AC-PEDOT ZIMCs achieve an areal capacity of 1.3 μAh/cm^2^, peak areal energy of 1.11 μWh/cm^2^, and peak areal power of 640 μW/cm^2^, surpassing most reported microsupercapacitors. This study highlights how optimized collectors and hybrid electrodes enhance microdevice charge storage while maximizing performance within a constrained footprint.

## Introduction

The rapid progress in wearable and implantable devices is revolutionizing technology, bringing them closer to becoming indispensable in our daily lives. These compact devices, excelling in complex tasks such as data processing and wireless communication within spaces smaller than half a square centimeter, hold transformative potential in health monitoring, medical diagnostics, and disease treatment.^1−3^ To sustain these devices, an essential component is an integrated on-chip energy storage unit, encompassing microbatteries and microsupercapacitors. While microbatteries offer high energy density, microsupercapacitors provide high power, rapid charging capabilities, and long cycle life.^4,5^ Certain applications, however, demand both high energy and high power density. Zinc-ion microcapacitors (ZIMCs) have emerged as promising candidates for these applications due to their intrinsic charge storage mechanism, which combines a battery-type anode with a capacitor-type cathode or vice versa.^6−8^ This hybrid configuration enables ZIMCs to achieve higher energy densities than microsupercapacitors and higher power densities than microbatteries, making them ideal for use in devices with limited footprints. Despite their potential, the development of ZIMCs is hindered by challenges in electrode material selection, microscale device design, and manufacturing complexity.

A critical factor influencing the performance of ZIMCs is the architecture of the current collectors and electrode materials. Traditional flat and planar interdigitated electrodes (IDEs), commonly used in ZIMCs, involve fabricating metal current collectors on the same substrate.^9,10^ While simple to fabricate, this approach often suffers from limited material utilization, reduced surface area, and sluggish ion transport, especially when integrating additional active materials, leading to suboptimal performance. Overcoming these limitations requires innovative strategies for electrode design and fabrication, particularly for on-chip applications, where both compactness and high performance are essential. However, the use of advanced IDEs and hybrid materials in ZIMCs remains underexplored, primarily due to difficulties in processing and incorporating these sophisticated materials. Hybrid electrode materials have demonstrated superior charge storage capabilities compared to their pristine counterparts due to synergistic interactions between components, which enhance overall storage capacity.^11,12^ These materials are widely employed in traditional energy storage devices, such as coin or pouch cells, where electrodes are arranged in a sandwich configuration with separators and electrolytes.^7^ However, their application in microscale energy storage devices, especially those utilizing planar designs with the cathode and anode printed on a single substrate in an IDE layout, remains largely untapped.

This study presents an advance design for high-performance ZIMCs aimed at addressing these challenges. It employs 3D gold (Au) IDEs as porous current collectors fabricated using a dynamic bubbling electrodeposition technique to produce a hierarchical porous structure with high conductivity and mechanical stability. The 3D Au IDEs are then loaded with zinc (Zn) as the anode material and hybrid activated carbon coated with PEDOT (AC-PEDOT) as the cathode. The fabrication process is executed using an advanced microplotter technique, which ensures precise material deposition and patterning, enabling uniform electrode formation and optimal material utilization.

## Results and Discussion

**Figure 1:**
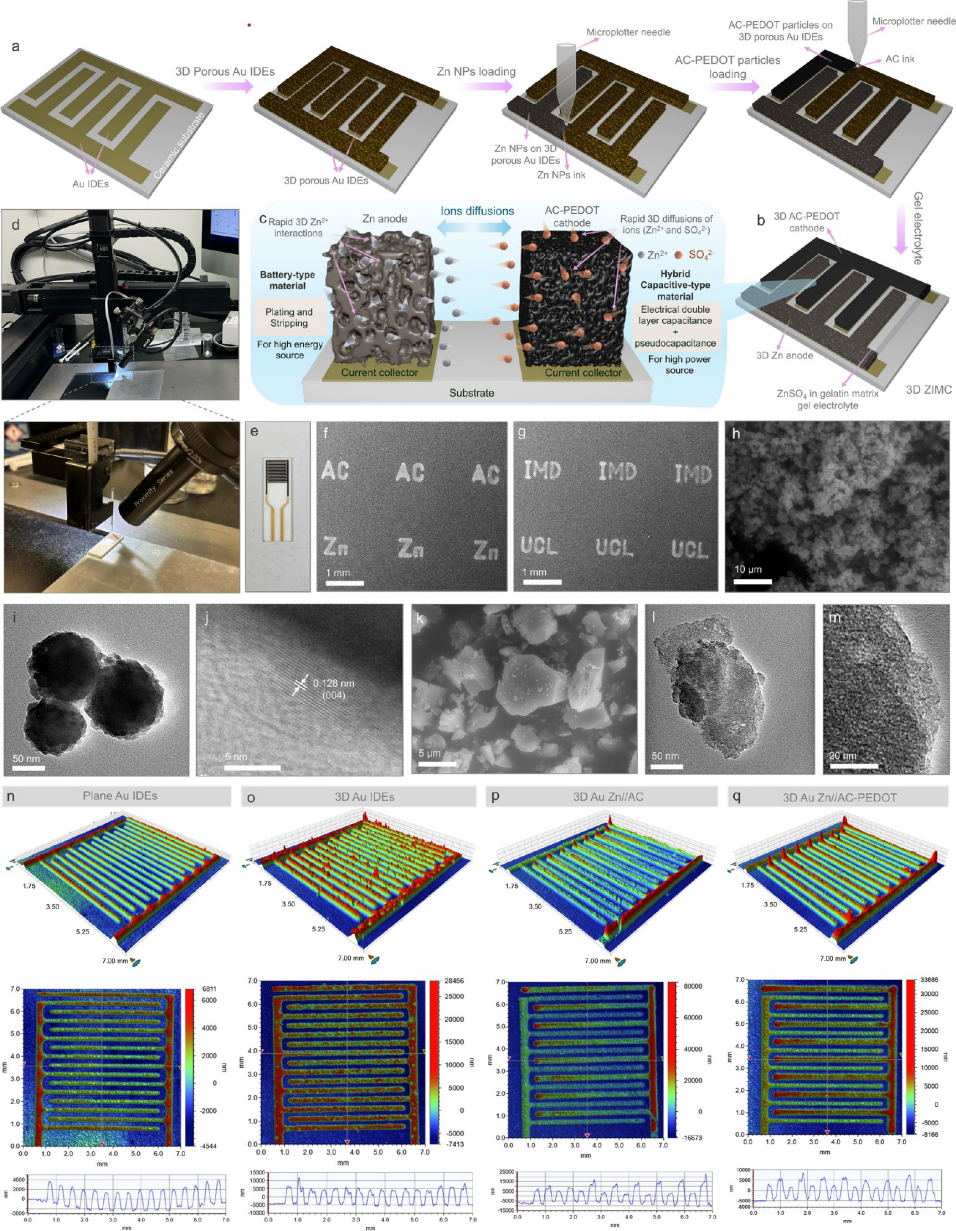
(a) Sequential stages in the fabrication of 3D Au Zn//AC-PEDOT ZIMCs, illustrating the patterning of Au IDEs with a 200 μm gap on a ceramic substrate, followed by the sequential loading of the Zn anode and AC cathode onto electrodeposited 3D porous Au using the microplotter technique followed by adding the PEDOT layer on AC through the electrodeposition technique. (b) Testing of the fabricated ZIMCs using a gel electrolyte. (c) Magnified view of a Zn//AC-PEDOT ZIMC, highlighting the in-plane diffusion of electrolyte ions for charge storage: battery-type storage on the Zn anode and hybrid type (EDLC and pseudocapacitive) storage on the AC-PEDOT cathode. (d) Digital images of the microplotter equipment. (e) Top-view digital images of a 3D Au Zn//AC-PEDOT ZIMC device. (f,g) SEM images of various directly written patterns on substrates. (h) SEM images of Zn nanopowders. (i,j) TEM images of Zn particles. (k) SEM images of AC particles. (l,m) TEM images of AC particles. 3D and 2D mappings and height profiles of the (n) plane Au IDE, (o) 3D Au IDE, (p) 3D Au Zn//AC ZIMC, and (q) 3D Au Zn//AC-PEDOT ZIMC.

The ZIMC fabrication process is illustrated in Figure 1a, while testing was conducted using a 1 M ZnSO_4_ gelatin-based electrolyte (Figure 1b). Preparation protocols for the inks and electrolyte are provided in the Methods. These planar ZIMCs exhibited an in-plane ion diffusion mechanism (Figure 1c), where the Zn anode displayed battery-like charge storage behavior through plating and stripping, delivering high energy storage. The AC-PEDOT cathode combined electrical double-layer capacitance (EDLC) with pseudocapacitive processes to achieve a high power output. The microplotter setup is shown in Figure 1d, and Figure 1e features a digital image of the fabricated 3D Au Zn//AC-PEDOT ZIMC device. Notably, the microplotter technique supports the processing of a wide range of materials and enables the creation of customizable patterns, including those on substrates with tailored designs such as letters (Figure 1f,g). These patterns can be precisely controlled through predesign on software, offering versatile and highly customizable fabrication capabilities.

Figure 1h–m provides fundamental characterizations of the raw materials, including SEM and TEM images of Zn nanopowders and AC powders. The SEM image of Zn nanopowder is shown in Figure 1h, while Figure 1i,j presents TEM images, revealing an average Zn particle size of approximately 100 nm and a (004) crystal phase with a *d*-spacing of 0.128 nm. The morphology of AC powders is depicted in Figure 1k, with TEM images in Figure 1l,m highlighting their amorphous structure. Figure 1n–q displays 3D and 2D mappings, along with height profiles, of various device configurations, including planar Au IDE, 3D Au IDE, 3D Au Zn//AC, and 3D Au//AC-PEDOT ZIMCs. Profilometry measurements indicate the thicknesses of the various layers within the 3D Au ZIMCs: 15 μm for the Zn anode, 20 μm for the AC cathode, 4 μm for the planar Au IDE, and 6 μm for the 3D porous Au layer.

**Figure 2:**
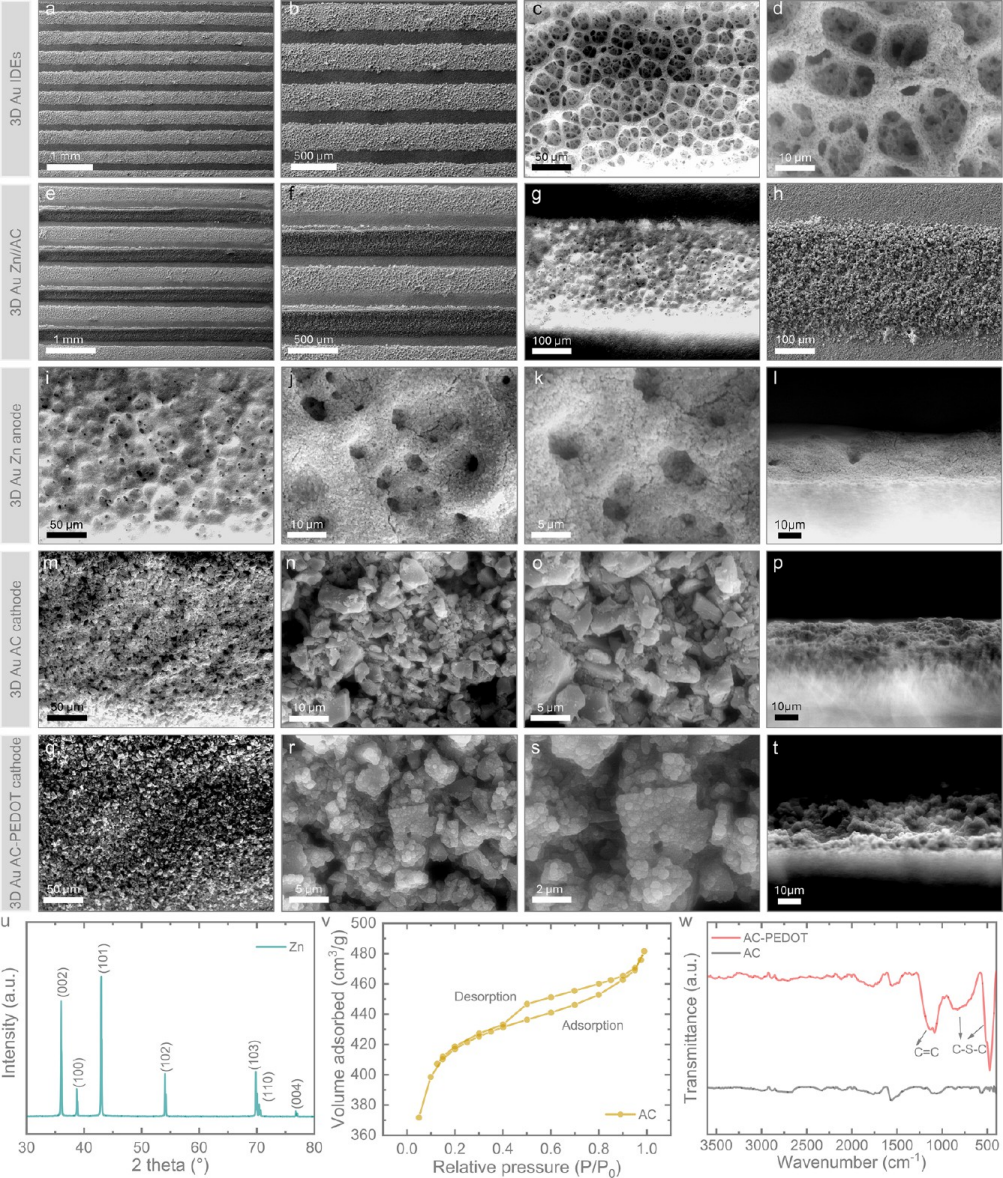
(a–d) SEM images of the 3D Au IDE at increasing magnifications. (e,f) SEM images of the 3D Au Zn//AC ZIMC. Low-magnification SEM images of the (g) Zn anode and the (h) AC cathode on the 3D Au IDE. SEM images of the Zn anode including (i–k) top views and (l) a cross-sectional view. SEM images of the AC cathode including (m–o) top views and (p) a cross-sectional view. SEM images of the AC-PEDOT cathode including (q–s) top views and (t) a cross-sectional view. (u) XRD pattern of Zn powder. (v) BET adsorption and desorption isotherms of N_2_ gas on the AC powder. (w) FTIR spectra of AC and AC-PEDOT.

SEM images in Figure 2a–d showcase the porous Au structure developed on planar Au IDEs at various magnifications, confirming the successful formation of a highly porous Au network via the dynamic bubbling electrodeposition technique.^3,13^Figure 2e–h illustrates the successful deposition of Zn and AC layers onto the 3D porous Au IDEs. High-magnification SEM images in Figure 2i–t provide detailed insights into the morphology of both electrode sides. Specifically, Figure 2i–k reveals Zn powder uniformly loaded onto the 3D Au structure, creating a porous architecture within the Zn anode. A cross-sectional SEM image in Figure 2l shows a Zn anode thickness of approximately 15 μm, consistent with the profilometry results shown in Figure 1p. Figure 2m–p compared with Figure 2q–t displays the uniform distribution of AC particles on the 3D Au IDE. The AC-PEDOT cathode images highlight spherical structures on the AC particles’ surfaces, attributed to PEDOT particles. The thickness of both the AC and AC-PEDOT cathodes, approximately 20 μm (Figure 2p,t), aligns with the measurements in Figure 1p,q. Detailed morphologies of the planar Zn//AC ZIMC are shown in Figure S1. Figure 2u presents the XRD pattern of Zn nanoparticles, showing characteristic peaks at 2θ = 36.0°, 38.7°, 43.0°, 54.1°, and 69.8°, corresponding to the (002), (100), (101), (102), and (103) planes of hexagonal zinc, as per PDF#87–0713. Additionally, Figure S2 shows AC peaks at 2θ = 22.1° and 43.5°, representing the (002) and (100) reflections, with peak broadening indicating an amorphous AC structure.^14^ Previous studies indicate PEDOT shows a broad XRD peak around 2θ = 25.6°, attributed to the π–π stacking of the PEDOT thiophene ring.^15^ A comparison of XRD patterns for AC and AC-PEDOT reveals broadening of the (002) peak in AC-PEDOT, likely due to the PEDOT coating on the AC surface. The AC used in this study demonstrates a high specific surface area of approximately 1586 m^2^/g, as determined by BET isotherm plots in Figure 2v. The FTIR spectra in Figure 2w confirm successful PEDOT coating on AC particles through electrodeposition, with characteristic peaks at ∼1105 cm^–1^ (C=C) and ∼825 and 476 cm^–1^ (C–S–C bonds).^16^

**Figure 3:**
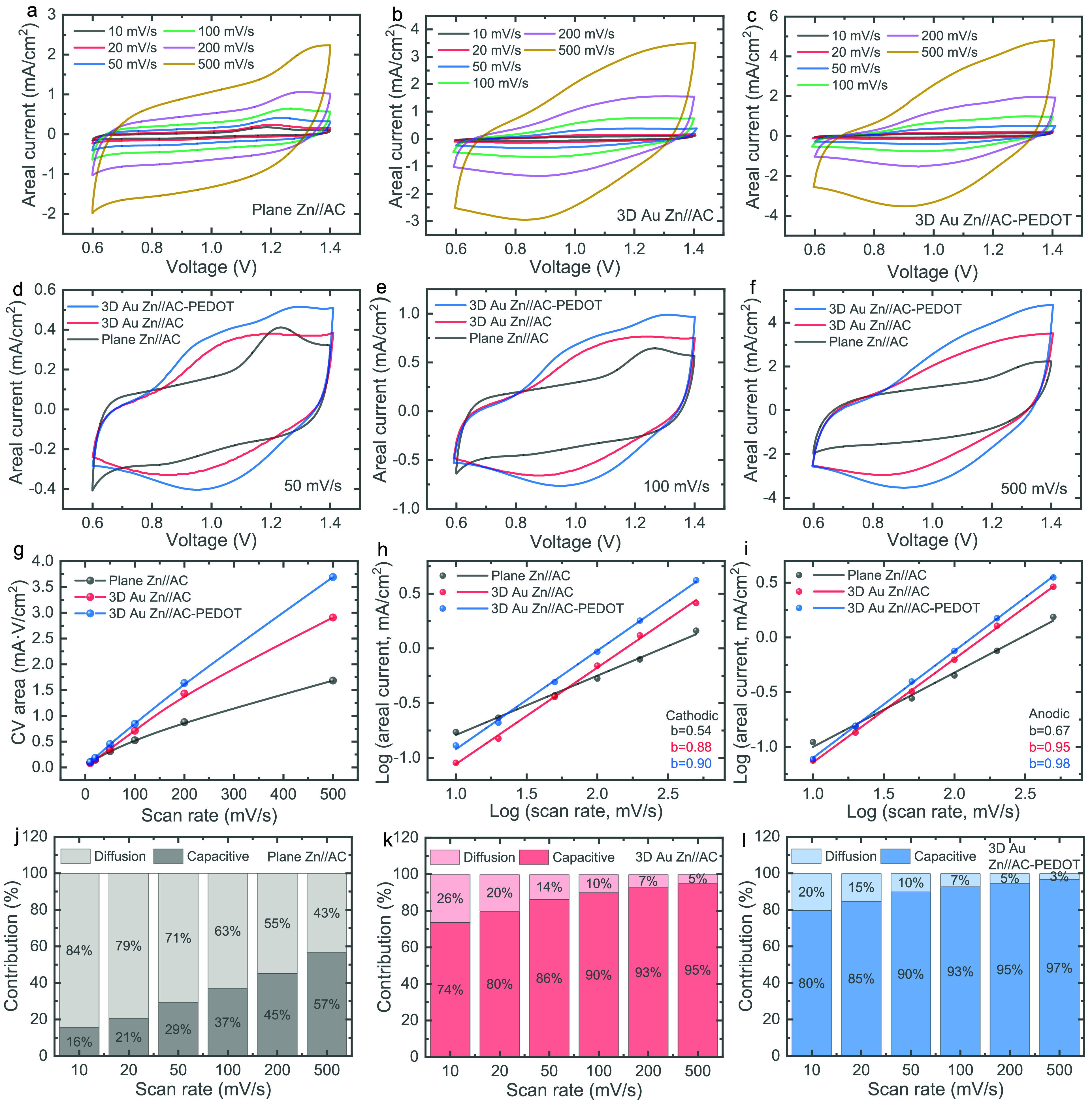
CV curves for (a) planar Zn//AC, (b) 3D Au Zn//AC, and (c) 3D Au Zn//AC-PEDOT ZIMCs across scan rates ranging from 10 to 500 mV/s. Comparative CV curves of the three ZIMCs at scan rates of (d) 50 mV/s, (e) 100 mV/s, and (f) 500 mV/s. (g) Plot of CV area versus scan rate for each ZIMC configuration. Determination of b values for the three ZIMCs from (h) cathodic and (i) anodic peaks. Capacitive- and diffusion-controlled charge storage contributions at various scan rates for (j) planar Zn//AC, (k) 3D Au Zn//AC, and (l) 3D Au Zn//AC-PEDOT ZIMCs.

We assessed the charge storage performance of the fabricated ZIMCs using a 1 M ZnSO_4_ gelatin gel electrolyte (Figure S3a). The ZIMC devices were immersed in a cuvette containing the gel electrolyte (Figure S3b), which was allowed to solidify for several hours before testing. Cyclic voltammetry (CV) tests were conducted at scan rates ranging from 10 mV/s to 500 mV/s within a voltage window of 0.6 to 1.4 V. Figure 3a–c presents the CV curves for the three different ZIMC configurations. When comparing CV curves at scan rates of 50 (Figure 3d), 100 (Figure 3e), and 500 mV/s (Figure 3f), the 3D Au Zn//AC ZIMCs exhibited a significant enhancement in charge storage performance, with a 72.8% increase in CV area at 500 mV/s compared to the planar Zn//AC ZIMCs. The 3D Au Zn//AC-PEDOT ZIMCs demonstrated an even greater improvement, with the CV area increasing by 119.3% and 26.9% relative to the planar Zn//AC and 3D Au Zn//AC ZIMCs, respectively, at 500 mV/s. The CV area for each scan rate across the three ZIMCs is summarized in Figure 3g. Additionally, we compared planar Zn//AC-PEDOT with 3D Au Zn//AC-PEDOT ZIMCs. The CV curves for planar Zn//AC-PEDOT, along with comparative CV curves at 200 and 500 mV/s, are presented in Figure S4. Notably, at 500 mV/s, the CV area increased by 20.9% for planar devices incorporating PEDOT (Zn//AC-PEDOT) and by 81.4% for 3D Au Zn//AC-PEDOT, highlighting the impact of the 3D Au structure. The overall charge storage mechanism can be classified into capacitive-controlled and diffusion-controlled processes, with the relationship between peak current (*i*) and scan rate (ν) expressed by *i* = aν^*b*^ or log *i* = *b* log ν + log a, where the *b* value near 0.5 suggests a diffusion-controlled electrochemical reaction, while a value near 1 indicates dominant capacitive behavior, where the *b* value is determined from the slope of log *i* vs log ν plots.^17^ The cathodic peak plots in Figure 3h display *b* values of 0.54, 0.88, and 0.90 for the planar Zn//AC, 3D Au Zn//AC, and 3D Au Zn//AC-PEDOT ZIMCs, respectively. Similarly, the anodic peak plots in Figure 3i reveal *b* values of 0.67, 0.95, and 0.98, respectively. These results suggest that the charge storage mechanism in 3D Au Zn//AC and 3D Au Zn//AC-PEDOT is predominantly governed by capacitive control, whereas planar Zn//AC exhibits charge storage dominated by diffusion control. Notably, the increase in capacitive-dominated charge storage facilitates operation at higher rates, confirming that the introduction of porous 3D Au IDEs significantly enhances the rate capability and overall charge storage performance (see further). Moreover, to quantify the contribution of both capacitive-controlled and diffusion-controlled processes to the overall charge storage performance, the current response (*i*) at a specific potential (*V*) can be expressed as *i*(*V*) = *k*_1_ν + *k*_2_ν^1/2^ or *i*(*V*)/ν^1/2^ = *k*_1_(*V*)ν^1/2^ + *k*_2_, where *k*_1_ν and *k*_2_ν^1/2^ correspond to capacitive- and diffusion-controlled contributions, respectively.^17^Figure 3j–l illustrates that the capacitive contributions in the three ZIMCs follow a trend similar to the previously calculated *b* values. The capacitive contribution increases with rising scan rates, with values of 16%, 74%, and 80% at 10 mV/s for planar Zn//AC, 3D Au Zn//AC, and 3D Au Zn//AC-PEDOT ZIMCs, respectively, which further increased to 57%, 95%, and 97% at a scan rate of 500 mV/s. These observations confirm the higher capacitive-controlled contribution in the 3D Au Zn//AC and 3D Au Zn//AC-PEDOT ZIMCs, attributed to the 3D porous Au IDEs structure that provides a large specific surface area with abundant active sites. In the case of the 3D Au Zn//AC-PEDOT ZIMC, the capacitive performance is further enhanced, likely due to the rapid pseudocapacitive behavior of PEDOT.^18^

**Figure 4:**
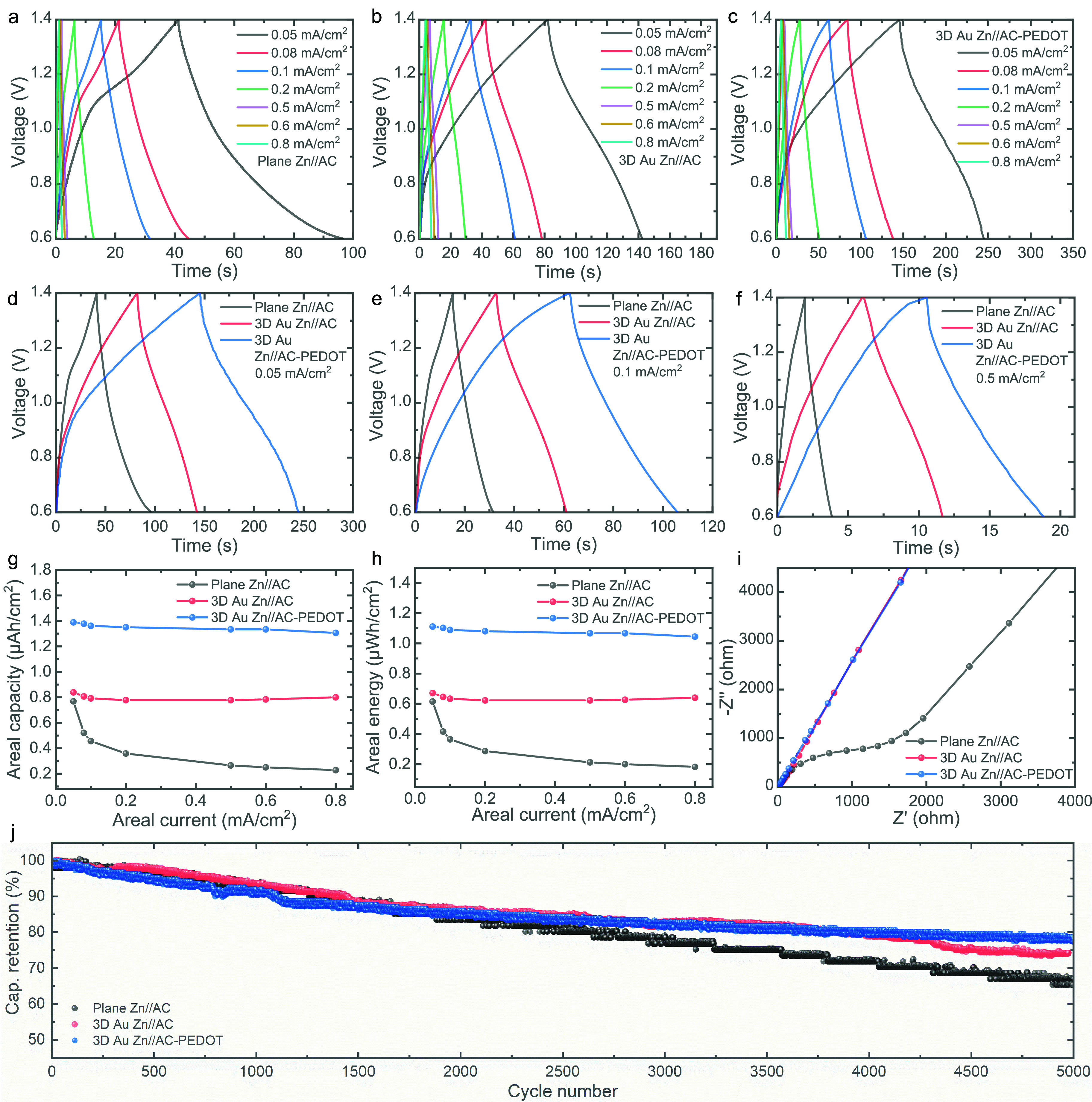
GCD profiles of (a) planar Zn//AC, (b) 3D Au Zn//AC, and (c) 3D Au Zn//AC-PEDOT ZIMCs at areal currents ranging from 0.05 to 0.8 mA/cm^2^. Comparative GCD profiles of the three ZIMCs at areal currents of (d) 0.05 mA/cm^2^, (e) 0.1 mA/cm^2^, and (f) 0.5 mA/cm^2^. (g) Areal capacity and (h) areal energy as a function of areal current. (i) Nyquist plots of the three ZIMCs obtained from EIS measurements. (j) Long-term cycling stability of the three ZIMCs over extended cycles.

To further investigate the charge storage capabilities of the ZIMCs, galvanostatic charge–discharge (GCD) tests were conducted at various areal currents ranging from 0.05 mA/cm^2^ to 0.8 mA/cm^2^, within the same voltage range (0.6 to 1.4 V) used in the CV analyses. The GCD profiles for planar Zn//AC, 3D Au Zn//AC, and 3D Au Zn//AC-PEDOT ZIMCs are presented in Figure 4a–c, respectively. Comparative GCD profiles at areal currents of 0.05, 0.1, and 0.5 mA/cm^2^ are displayed in Figure 4d–f, respectively, revealing superior charge storage performance for both 3D Au Zn//AC and 3D Au Zn//AC-PEDOT ZIMCs compared to planar Zn//AC ZIMCs, consistent with the CV results. The enhanced charge storage performance observed with the introduction of porous 3D Au IDEs is likely due to their synergistic effects, which include reduced charge transfer resistance, improved conductivity, and efficient loading of active materials onto the porous Au IDEs without compromising charge transfer kinetics. Additionally, the incorporation of PEDOT on the AC cathode improves the charge storage performance of the 3D Au Zn//AC-PEDOT ZIMCs. This enhancement is likely due to the pseudocapacitive properties of PEDOT, which contribute to a hybrid charge storage mechanism that combines pseudocapacitive behavior with an electrical double-layer charge storage mechanism within the AC-PEDOT composite. Both 3D Au ZIMCs exhibit stable GCD profiles even at higher areal currents (1 to 10 mA/cm^2^), as shown in Figure S5. The plot of areal capacity and areal energy versus areal current (Figure 4g,h) demonstrates substantial performance improvements in 3D Au ZIMCs across all of the tested areal currents. For example, the areal capacities of planar Zn//AC ZIMCs at 0.2, 0.5, and 0.8 mA/cm^2^ were measured at 0.36, 0.26, and 0.23 μAh/cm^2^, respectively. In contrast, the corresponding values for 3D Au Zn//AC ZIMCs increased to 0.79, 0.78, and 0.8 μAh/cm^2^, while 3D Au Zn//AC-PEDOT ZIMCs achieved even higher capacities of 1.35, 1.33, and 1.31 μAh/cm^2^. Additionally, GCD tests were conducted on planar Zn//AC-PEDOT, as shown in Figure S6. The results demonstrate that incorporating PEDOT improves the areal capacity even in planar Zn//AC-PEDOT, while the integration of 3D porous Au IDEs significantly enhances charge storage performance in 3D Au Zn//AC-PEDOT. It is evident that the capacity drops significantly with increasing areal current in planar Zn//AC ZIMCs, while both 3D Au Zn//AC and 3D Au Zn//AC-PEDOT ZIMCs maintain superior performance. This enhancement is attributed to the capacitive-dominated charge storage enabled by the introduction of 3D Au IDEs, as previously discussed, resulting in superior rate performance. Furthermore, Figure 4h highlights a notable improvement in areal energy for 3D Au Zn//AC-PEDOT ZIMCs, achieving 1.04 μWh/cm^2^ at 0.8 mA/cm^2^ compared to only 0.18 μWh/cm^2^ for planar Zn//AC ZIMCs.

To gain deeper insight into the charge storage kinetics of the ZIMCs, electrochemical impedance spectroscopy (EIS) was performed, and the results are shown as Nyquist plots in Figure 4i. The enlarged Nyquist plots of the three ZIMCs in the high-frequency region are presented in Figure S7. The 3D Au ZIMCs show significantly lower equivalent series resistance, with values of 7.18 ohms for 3D Au Zn//AC and 6.49 ohms for 3D Au Zn//AC-PEDOT, compared to 14.12 ohms for planar Zn//AC ZIMCs, as shown in Figure S8. The fitted Nyquist plots further confirm that after the introduction of 3D Au IDEs, the semicircle associated with charge transfer resistance becomes almost negligible, whereas the planar Zn//AC ZIMC displays a distinct charge transfer resistance of 1220 ohms (Figure S8). These observations are consistent with the *b* value analysis (Figure 3h,i) and the capacitive- and diffusion-controlled contributions (Figure 3j–l). The introduction of 3D Au IDEs promotes a capacitive-controlled charge storage mechanism, significantly enhancing overall charge storage, while the planar Zn//AC ZIMC exhibits a diffusion-controlled charge storage mechanism, resulting in slower charge transfer and reduced performance.

**Figure 5:**
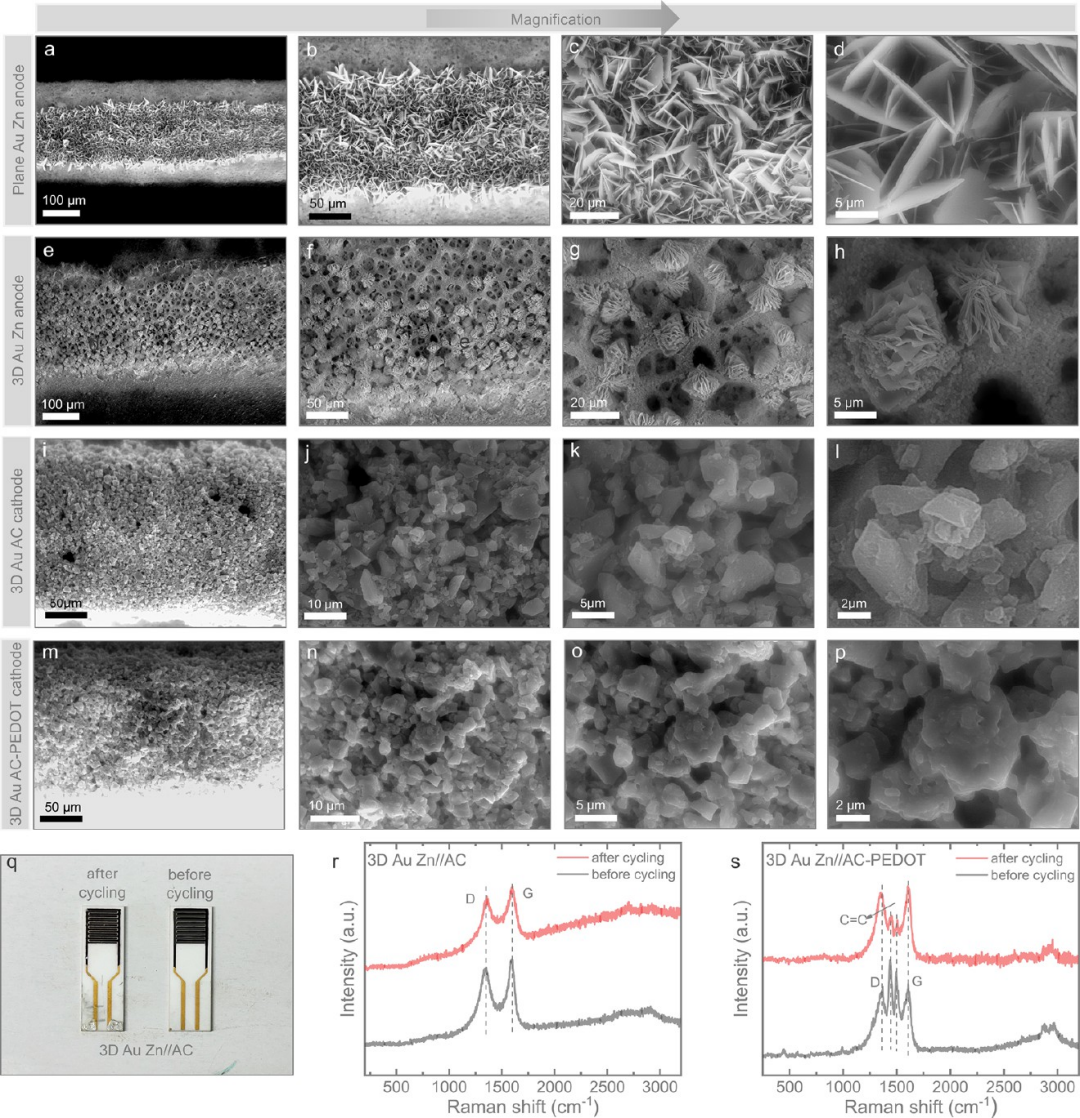
SEM images after 5000 cycles: (a–d) plane Zn anode, (e–h) 3D Au Zn anode, (i–l) 3D Au AC cathode, and (m–p) 3D Au AC-PEDOT cathode. (q) Digital image of 3D Au Zn//AC before and after cycling. Raman spectra of the (r) 3D Au AC and (s) 3D Au AC-PEDOT cathode before and after cycling.

To evaluate the long-term cycling stability of the ZIMCs, extended cycling tests were conducted, as shown in Figure 4j. It was observed that ZIMCs with 3D Au IDEs not only demonstrated superior charge storage performance but also exhibited enhanced cycling stability, with the capacity retention after 5000 cycles recorded at 67%, 74.3%, and 78% for planar Zn//AC, 3D Au Zn//AC, and 3D Au Zn//AC-PEDOT ZIMCs, respectively. In general, AC materials exhibit superior cycling stability compared to conducting polymers like PEDOT due to their distinct charge storage mechanisms. While AC primarily relies on electrical double-layer charge storage, PEDOT contributes through pseudocapacitive charge storage. Micropatterned electrode materials, fabricated using inks, introduce contact resistance between the current collectors and electrode materials, leading to increased charge transfer resistance. However, electrodeposition of PEDOT onto AC materials effectively reduces interfacial resistance by suppressing charge transfer resistance. Furthermore, when AC particles are incorporated into the porous Au scaffold, a substantial portion infiltrates the interior of the porous network, enhancing the interfacial charge transfer. The subsequent electrodeposition of PEDOT improves the particle-to-particle contact, ultimately leading to better long-term cycling stability. Thus, the 3D Au Zn//AC-PEDOT ZIMC exhibits enhanced cycling stability and durability compared to the planar Zn//AC ZIMC. After 5000 cycles, the ZIMCs were disassembled, and the electrolyte was cleaned to analyze the morphology and stability of the microelectrodes within the IDEs (Figure 5a–p presents SEM images of the three ZIMCs at varying magnifications). Digital images of the electrodes are shown in Figure 5q. On the planar Zn anode, the morphology of Zn nanoparticles drastically transformed into sharp, flaky structures (Figure 5d). In contrast, the 3D Au Zn anode retained flower-like flaky morphologies with porous Au structures appearing at certain points on the electrode (Figure 5h). Meanwhile, no significant morphological changes were observed on the 3D Au AC and AC-PEDOT cathodes after cycling, as expected based on their charge storage characteristics. Additionally, Raman spectra (Figure 5r,s) revealed a slight increase in the intensity ratio of the D (∼1353 cm^–1^) to G (∼1596 cm^–1^) bands (I_*D*_/I_*G*_), rising from 0.91 to 0.94 for the 3D Au AC cathode and from 0.91 to 0.96 for the 3D Au AC-PEDOT cathode (the electrolyte was cleaned). This increase suggests a slight increase in structural disorder or defect density in the AC cathode materials after prolonged cycling. Peaks at approximately 1438 and 1495 cm^–1^ in Figure 5s correspond to the C=C symmetric and asymmetric stretching in PEDOT.^19,20^ The reduced intensity of these peaks postcycling may be due to signal suppression by the gel electrolyte matrix, as complete removal of the electrolyte is challenging for ex situ characterization. However, the presence of these peaks confirms that PEDOT remains intact, even after extensive cycling of the 3D Au Zn//AC-PEDOT ZIMCs microcathode.

**Figure 6:**
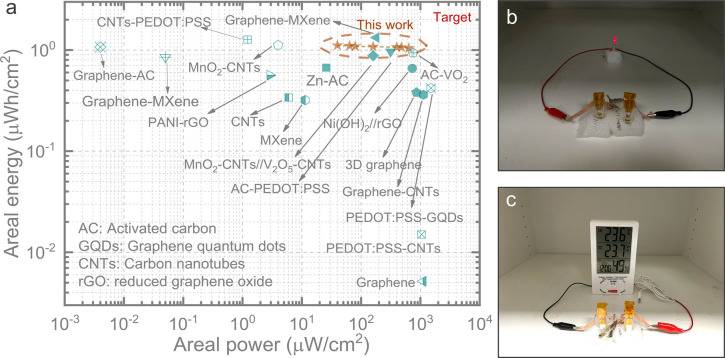
(a) Ragone plot comparing the performance of 3D Au Zn//AC-PEDOT ZIMCs with previously reported microsupercapacitors including both symmetric and asymmetric designs as well as hybrid capacitors: graphene,^29^ PEDOT:PSS-CNTs,^21^ 3D graphene,^22^ MXene,^30^ CNTs,^24^ graphene-CNTs,^23^ MnO_2_–CNTs//V_2_O_5_–CNTs,^26^ Ni(OH)_2_//rGO,^27^ AC-PEDOT:PSS^15^ , graphene-MXene,^31,32^ CNTs-PEDOT:PSS,^33^ PEDOT:PSS-GQDs,^25^ PANI-rGO,^34^ graphene-AC,^35^ MnO_2_–CNTs,^36^ Zn//AC,^28^ and AC//VO_2_^10^. (b) Demonstration of two ZIMCs connected in series to power an LED. (c) Demonstration of two ZIMCs connected in series to power a thermohygrometer.

Additionally, we calculated the areal energies and areal powers of the 3D Au Zn//AC and 3D Au Zn//AC-PEDOT ZIMCs. Specifically, the areal energies for 3D Au Zn//AC ZIMCs were 0.67 μWh/cm^2^ at 40 μW/cm^2^, 0.63 μWh/cm^2^ at 80 μW/cm^2^, 0.62 μWh/cm^2^ at 400 μW/cm^2^, and 0.64 μWh/cm^2^ at 640 μW/cm^2^. For the 3D Au Zn//AC-PEDOT ZIMCs, the areal energies were 1.11 μWh/cm^2^ at 40 μW/cm^2^, 1.09 μWh/cm^2^ at 80 μW/cm^2^, 1.07 μWh/cm^2^ at 400 μW/cm^2^, and 1.04 μWh/cm^2^ at 640 μW/cm^2^. These values surpass the performance of numerous previously reported high-performance symmetric and asymmetric microsupercapacitors as well as hybrid capacitors, as shown in Figure 6a. For example, they outperform systems such as PEDOT:PSS-CNTs (0.015 μWh/cm^2^ at 1050 μW/cm^2^),^21^ 3D graphene (0.38 μWh/cm^2^ at 860 μW/cm^2^),^22^ graphene-CNTs (0.361 μWh/cm^2^ at 1130 μW/cm^2^),^23^ CNTs (0.34 μWh/cm^2^ at 6 μW/cm^2^),^24^ and PEDOT:PSS-GQDs (0.42 μWh/cm^2^ at 1510 μW/cm^2^)^25^ based symmetric microsupercapacitors. Additionally, the performance also exceeds that of asymmetric systems, including MnO_2_–CNTs//V_2_O_5_–CNTs (0.88 μWh/cm^2^ at 160 μW/cm^2^),^26^ Ni(OH)_2_//rGO (0.66 μWh/cm^2^ at 730 μW/cm^2^),^27^ AC//VO_2_ (0.94 μWh/cm^2^ at 753.12 μW/cm^2^),^10^ and Zn//AC (0.67 μWh/cm^2^ at 25.87 μW/cm^2^).^28^ Furthermore, the integration of two 3D Au Zn//AC ZIMCs in series demonstrated their practical application by successfully powering an LED and a thermohygrometer, as depicted in Figure 6b,c.

## Conclusions

This study demonstrates the immense potential of ZIMCs as high-performance energy storage solutions for microscale applications. By integrating 3D Au IDEs as porous current collectors and leveraging hybrid AC-PEDOT as the cathode, we address the critical challenges of electrode material selection and microscale device design. The advanced structure of the 3D Au IDEs significantly enhances charge storage by increasing the surface area, facilitating ion diffusion, and improving overall conductivity. Coupled with the advanced microplotter fabrication technique, this approach ensures precise material deposition and uniform electrode formation. The resulting 3D Au Zn//AC-PEDOT ZIMCs exhibit a remarkable areal capacity of 1.3 μAh/cm^2^, a peak areal energy of 1.11 μWh/cm^2^, and a peak areal power of 640 μW/cm^2^, outperforming most previously reported microsupercapacitors and microcapacitors. These improvements are attributed to the synergistic effects of the hybrid materials and the optimized electrode architecture, which collectively boost capacitive-controlled charge storage and long-term cycling stability. This work highlights the importance of innovative electrode design and material integration in advancing ZIMC technology, paving the way for high-performance, on-chip energy storage devices.

## Methods

### Materials

Zinc nanoparticles with an average size ranging from 40 to 150 nm were procured from Sigma-Aldrich. Activated carbon powder was sourced from MTI Corporation, and triethylene glycol monomethyl ether (TEGMME) was supplied by Sigma-Aldrich. Gold(III) chloride trihydrate (AuCl_4_·3H_2_O) and ammonium chloride (NH_4_Cl), both obtained from Sigma-Aldrich, were used in the fabrication of porous gold. All chemicals were used as received, without any further purification. 3,4-Ethylenedioxythiophene (EDOT), sodium dodecyl sulfate (SDS), and sulfuric acid (H_2_SO_4_) are utilized as components for the electrochemical deposition of PEDOT.

### Electrodeposition of 3D Porous Au

The commercial plane Au IDE with 200 μm lines and gaps (DRP-IDEAU200), sourced from Metrohm U.K. Ltd., was cleaned prior to use with isopropanol, followed by distilled water, and dried using nitrogen gas. To prepare the deposition solution, a mixture of 0.1 M gold(III) chloride trihydrate (AuCl_4_·3H_2_O) and 2 M ammonium chloride (NH_4_Cl) was dissolved in 20 mL of distilled water. This solution was used to electrodeposit porous gold onto the flat IDEs by using the dynamic bubble method. To maintain consistency, the solution was continuously stirred for one hour at room temperature. A platinum wire (Pt) and a Ag/AgCl electrode were employed as the counter and reference electrodes, respectively. Electrodeposition of porous gold onto the flat IDE devices was carried out at a voltage of −2 V for 5 s. After electrodeposition, the devices were rinsed with distilled water and allowed to dry before use. The electrodeposition process was conducted by using an Autolab electrochemical workstation.

### Preparation of Electrode Inks

In a vessel, 4 g of triethylene glycol monomethyl ether (TEGMME) and 6 g of zinc nanopowder were combined, and the mixture was subjected to sonication for 30 min using a Fisherbrand 505 sonicator. The sonicator was set to an amplitude of 30% with a pulse configuration of 01 01. Similarly, for the preparation of the activated carbon ink, 4 g of TEGMME and 2.5 g of activated carbon were sonicated for 30 min under the same settings.

### Printing of ZIMCs

The zinc and activated carbon inks were applied onto 3D Au IDEs prepared by the electrodeposition mentioned in the previous paragraph, using a SonoPlot Microplotter Proto, equipped with a 20 μm nozzle. Prior to the printing process, the ZIMC pattern was designed by using SonoDraw software. During the printing process, the nozzle was aligned with the starting point of the pattern and the feature width was set to 50 μm. The printing voltage was set to 8 V for the zinc ink and 12 V for the activated carbon ink with the spraying mode employed for both inks.

### Electrodeposition of PEDOT on the AC Cathode

A 0.01 M sodium dodecyl sulfate aqueous solution was prepared, and concentrated H_2_SO_4_ was added until the final concentration of H_2_SO_4_ reached 1 M. Ethylene dioxythiophene (EDOT) was then introduced into the solution with continuous stirring until the solution turned slightly blue, signaling the formation of the electrolyte. The PEDOT layer was subsequently electrochemically deposited onto the AC cathode by applying a potential of 0.9 V for 200 s.

### Material Characterizations

XRD measurements were conducted using an AERIS PANalytical Research Edition system with Cu Kα radiation (λ = 0.154 nm) at 40 kV and 7.5 mA, with a scanning range of 10° to 80° at a scan rate of 5° per minute. Raman spectroscopy was performed with a Renishaw inVia confocal Raman microscope, utilizing a 532 nm laser covering a range from 100 to 3200 cm^–1^. The morphology of the devices was examined using a scanning electron microscope (SEM, Zeiss EVO LS15), while transmission electron microscopy (TEM, JEOL 2100) was employed for higher-magnification imaging to analyze the powder structure. Fourier transform infrared (FT-IR) spectroscopy was carried out using a PerkinElmer Spectrum Two spectrometer to investigate the functional groups in the powders. The specific surface area of the activated carbon was measured using a NOVAtouch BET instrument. Additionally, the surface profile of the devices was analyzed with a stylus profilometer (Bruker DektakXT).

### Electrochemical Testing

To evaluate the performance of the developed microsupercapacitors, 1 M ZnSO_4_ gel electrolyte was prepared by dissolving 2 g of gelatin into 15 mL of 1 M ZnSO_4_ electrolyte at 80 °C. The two ends of the electrodeposited IDE devices were connected to copper foil using silver paste. The devices were then vertically immersed in an electrolyte-filled cuvette (Fisherbrand Disposable Cuvettes 14955125), and the top of the cuvette was sealed with parafilm. The assembled devices were interfaced with an electrochemical tester (Biologic) for CV measurements, conducted at scan rates ranging from 10 to 500 mV/s within a 0.6–1.4 V voltage window. GCD tests were performed at current densities ranging from 0.05 to 10 mA/cm^2^. Long-term cycling stability was assessed using a Neware battery tester under an areal current of 0.1 mA/cm^2^ for 5000 cycles. Electrochemical impedance spectroscopy (EIS) measurements were conducted with an IVIUM electrochemical workstation, operating over a frequency range of 10 mHz to 100 kHz with a 10 mV voltage amplitude at OCV.

## Supporting Information Available

SEM images of the Zn//AC ZIMC; XRD patterns of samples; digital images of the electrolyte and ZIMC immersed in the electrolyte; CVs and GCDs of ZIMCs; and Nyquist plots and equivalent circuits analysis of ZIMCs (PDF)

## References

[ref1] Li P.; Liao M.; Li J.; Ye L.; Cheng X.; Wang B.; Peng H. Rechargeable Micro-Batteries for Wearable and Implantable Applications. Small Struct. 2022, 3 (9), 2200058 10.1002/sstr.202200058.

[ref2] Xia Q.; Zan F.; Zhang Q.; Liu W.; Li Q.; He Y.; Hua J.; Liu J.; Xu J.; Wang J.; Wu C.; Xia H. All-Solid-State Thin Film Lithium/Lithium-Ion Microbatteries for Powering the Internet of Things. Adv. Mater. 2023, 35 (2), 2200538 10.1002/adma.202200538. 35962983

[ref3] Naresh N.; Zhu Y.; Fan Y.; Luo J.; Wang T.; Parkin I. P.; Boruah B. D. Advanced Porous Gold-PANI Micro-Electrodes for High-Performance On-Chip Micro-Supercapacitors. Nano Lett. 2024, 24 (35), 11059–11066. 10.1021/acs.nanolett.4c03194. 39186689 PMC11378337

[ref4] Zhang J.; Zhang G.; Zhou T.; Sun S. Recent Developments of Planar Micro-Supercapacitors: Fabrication, Properties, and Applications. Adv. Funct. Mater. 2020, 30 (19), 1910000 10.1002/adfm.201910000.

[ref5] Zhang P.; Yang S.; Xie H.; Li Y.; Wang F.; Gao M.; Guo K.; Wang R.; Lu X. Advanced Three-Dimensional Microelectrode Architecture Design for High-Performance On-Chip Micro-Supercapacitors. ACS Nano 2022, 16 (11), 17593–17612. 10.1021/acsnano.2c07609. 36367555

[ref6] Tang H.; Yao J.; Zhu Y. Recent Developments and Future Prospects for Zinc-Ion Hybrid Capacitors: A Review. Adv. Energy Mater. 2021, 11 (14), 2003994 10.1002/aenm.202003994.

[ref7] Liu Y.; Wu L. Recent Advances of Cathode Materials for Zinc-Ion Hybrid Capacitors. Nano Energy 2023, 109, 108290 10.1016/j.nanoen.2023.108290.

[ref8] George N. S.; Singh G.; Bahadur R.; Kumar P.; Ramadass K.; Sathish C.; Benzigar M.; Sajan D.; Aravind A.; Vinu A. Recent Advances in Functionalized Biomass-Derived Porous Carbons and Their Composites for Hybrid Ion Capacitors. Advanced Science 2024, 2406235 10.1002/advs.202406235. 39031008 PMC11425278

[ref9] Zhang P.; Li Y.; Wang G.; Wang F.; Yang S.; Zhu F.; Zhuang X.; Schmidt O. G.; Feng X. Zn-Ion Hybrid Micro-Supercapacitors with Ultrahigh Areal Energy Density and Long-Term Durability. Adv. Mater. 2019, 31 (3), 1806005 10.1002/adma.201806005. 30480352

[ref10] Fan Y.; Pinnock I.; Hu X.; Wang T.; Lu Y.; Li R.; Wang M.; Parkin I. P.; De Volder M.; Boruah B. D. Planar Zn-Ion Microcapacitors with High-Capacity Activated Carbon Anode and VO _2_ (B) Cathode. Nano Lett. 2024, 24 (35), 10874–10882. 10.1021/acs.nanolett.4c02539. 39163512 PMC11378291

[ref11] Javed M. S.; Asim S.; Najam T.; Khalid M.; Hussain I.; Ahmad A.; Assiri M. A.; Han W. Recent Progress in Flexible Zn-ion Hybrid Supercapacitors: Fundamentals, Fabrication Designs, and Applications. Carbon Energy 2023, 5 (1), e271 10.1002/cey2.271.

[ref12] Wang Y.; Sun S.; Wu X.; Liang H.; Zhang W. Status and Opportunities of Zinc Ion Hybrid Capacitors: Focus on Carbon Materials, Current Collectors, and Separators. Nanomicro Lett. 2023, 15 (1), 78 10.1007/s40820-023-01065-x. 36988736 PMC10060505

[ref13] Naresh N.; Zhu Y.; Luo J.; Fan Y.; Wang T.; Raju K.; De Volder M.; Parkin I. P.; Boruah B. D. Advanced 3D Micro-Electrodes for On-Chip Zinc-Ion Micro-Batteries. Adv. Funct. Mater. 2025, 35, 2413777 10.1002/adfm.202413777.

[ref14] Wang S.; Nam H.; Gebreegziabher T. B.; Nam H. Adsorption of Acetic Acid and Hydrogen Sulfide Using NaOH Impregnated Activated Carbon for Indoor Air Purification. Eng. Rep. 2020, 2 (1), e12083 10.1002/eng2.12083.

[ref15] Fan Y.; Wang T.; Asrosa R.; Li B.; Naresh N.; Liu X.; Guan S.; Li R.; Wang M.; Parkin I. P.; Boruah B. D. Synergistic Contribution of Activated Carbon and PEDOT:PSS in Hybrid Electrodes for High-Performance Planar Micro-Supercapacitors. Chem. Eng. J. 2024, 488, 150672 10.1016/j.cej.2024.150672.

[ref16] Ly C. T.; Phan C. T.; Vu C. N.; Le H. S.; Nguyen T. T.; Tran D. L.; Le L. A.; Vu T. T. Electrodeposition of PEDOT-RGO Film in Aqueous Solution for Detection of Acetaminophen in Traditional Medicaments. Adv. Nat. Sci.:Nanosci. Nanotechnol. 2019, 10 (1), 015013 10.1088/2043-6254/ab0883.

[ref17] Deka Boruah B.; Mathieson A.; Park S. K.; Zhang X.; Wen B.; Tan L.; Boies A.; De Volder M. Vanadium Dioxide Cathodes for High-Rate Photo-Rechargeable Zinc-Ion Batteries. Adv. Energy Mater. 2021, 11 (13), 2100115 10.1002/aenm.202100115.

[ref18] Adekoya G. J.; Sadiku R. E.; Hamam Y.; Ray S. S.; Mwakikunga B. W.; Folorunso O.; Adekoya O. C.; Lolu O. J.; Biotidara O. F. Pseudocapacitive Material for Energy Storage Application: PEDOT and PEDOT:PSS AIP Conference Proceedings, 2020, p 020073. 10.1063/5.0028340.

[ref19] Tzaneva B.; Mateev V.; Stefanov B.; Aleksandrova M.; Iliev I. Electrochemical Investigation of PEDOT:PSS/Graphene Aging in Artificial Sweat. Polymers 2024, 16 (12), 1706 10.3390/polym16121706. 38932055 PMC11207453

[ref20] Xiong S.; Zhang L.; Lu X. Conductivities Enhancement of Poly(3,4-Ethylenedioxythiophene)/Poly(Styrene Sulfonate) Transparent Electrodes with Diol Additives. Polym. Bull. 2013, 70 (1), 237–247. 10.1007/s00289-012-0833-8.

[ref21] Liu W.; Lu C.; Li H.; Tay R. Y.; Sun L.; Wang X.; Chow W. L.; Wang X.; Tay B. K.; Chen Z.; Yan J.; Feng K.; Lui G.; Tjandra R.; Rasenthiram L.; Chiu G.; Yu A. Paper-Based All-Solid-State Flexible Micro-Supercapacitors with Ultra-High Rate and Rapid Frequency Response Capabilities. J. Mater. Chem. A 2016, 4 (10), 3754–3764. 10.1039/C6TA00159A.

[ref22] Zhang L.; DeArmond D.; Alvarez N. T.; Malik R.; Oslin N.; McConnell C.; Adusei P. K.; Hsieh Y.; Shanov V. Flexible Micro-Supercapacitor Based on Graphene with 3D Structure. Small 2017, 13 (10), 1603114 10.1002/smll.201603114. 28054423

[ref23] Bellani S.; Petroni E.; Del Rio Castillo A. E.; Curreli N.; Martín-García B.; Oropesa-Nuñez R.; Prato M.; Bonaccorso F. Scalable Production of Graphene Inks via Wet-Jet Milling Exfoliation for Screen-Printed Micro-Supercapacitors. Adv. Funct. Mater. 2019, 29 (14), 1807659 10.1002/adfm.201807659.

[ref24] Kim H.; Yoon J.; Lee G.; Paik S.; Choi G.; Kim D.; Kim B.-M.; Zi G.; Ha J. S. Encapsulated, High-Performance, Stretchable Array of Stacked Planar Micro-Supercapacitors as Waterproof Wearable Energy Storage Devices. ACS Appl. Mater. Interfaces 2016, 8 (25), 16016–16025. 10.1021/acsami.6b03504. 27267316

[ref25] Li Z.; Ruiz V.; Mishukova V.; Wan Q.; Liu H.; Xue H.; Gao Y.; Cao G.; Li Y.; Zhuang X.; Weissenrieder J.; Cheng S.; Li J. Inkjet Printed Disposable High-Rate On-Paper Microsupercapacitors. Adv. Funct. Mater. 2022, 32 (1), 2108773 10.1002/adfm.202108773.

[ref26] Yun J.; Lim Y.; Lee H.; Lee G.; Park H.; Hong S. Y.; Jin S. W.; Lee Y. H.; Lee S.; Ha J. S. A Patterned Graphene/ZnO UV Sensor Driven by Integrated Asymmetric Micro-Supercapacitors on a Liquid Metal Patterned Foldable Paper. Adv. Funct. Mater. 2017, 27 (30), 1700135 10.1002/adfm.201700135.

[ref27] Huang G.-W.; Li N.; Du Y.; Feng Q.-P.; Xiao H.-M.; Wu X.-H.; Fu S.-Y. Laser-Printed In-Plane Micro-Supercapacitors: From Symmetric to Asymmetric Structure. ACS Appl. Mater. Interfaces 2018, 10 (1), 723–732. 10.1021/acsami.7b15922. 29243912

[ref28] Fan Y.; Liu X.; Naresh N.; Zhu Y.; Pinnock I.; Wang T.; Wang M.; Parkin I. P.; Boruah B. D. High-Performance Planar Zn-Ion Micro-Capacitors. J. Mater. Chem. A 2024, 12 (19), 11710–11718. 10.1039/D4TA00300D.

[ref29] Li L.; Secor E. B.; Chen K.; Zhu J.; Liu X.; Gao T. Z.; Seo J. T.; Zhao Y.; Hersam M. C. High-Performance Solid-State Supercapacitors and Microsupercapacitors Derived from Printable Graphene Inks. Adv. Energy Mater. 2016, 6 (20), 1600909 10.1002/aenm.201600909.

[ref30] Zhang C.; McKeon L.; Kremer M. P.; Park S.-H.; Ronan O.; Seral-Ascaso A.; Barwich S.; Coileáin C. O. ´.; McEvoy N.; Nerl H. C.; Anasori B.; Coleman J. N.; Gogotsi Y.; Nicolosi V. Additive-Free MXene Inks and Direct Printing of Micro-Supercapacitors. Nat. Commun. 2019, 10 (1), 1795 10.1038/s41467-019-09398-1. 30996224 PMC6470171

[ref31] Yue Y.; Liu N.; Ma Y.; Wang S.; Liu W.; Luo C.; Zhang H.; Cheng F.; Rao J.; Hu X.; Su J.; Gao Y. Highly Self-Healable 3D Microsupercapacitor with MXene–Graphene Composite Aerogel. ACS Nano 2018, 12 (5), 4224–4232. 10.1021/acsnano.7b07528. 29648800

[ref32] Li H.; Hou Y.; Wang F.; Lohe M. R.; Zhuang X.; Niu L.; Feng X. Flexible All-Solid-State Supercapacitors with High Volumetric Capacitances Boosted by Solution Processable MXene and Electrochemically Exfoliated Graphene. Adv. Energy Mater. 2017, 7 (4), 1601847 10.1002/aenm.201601847.

[ref33] Xiao H.; Wu Z.-S.; Zhou F.; Zheng S.; Sui D.; Chen Y.; Bao X. Stretchable Tandem Micro-Supercapacitors with High Voltage Output and Exceptional Mechanical Robustness. Energy Storage Mater. 2018, 13, 233–240. 10.1016/j.ensm.2018.01.019.

[ref34] Park S.; Lee H.; Kim Y.-J.; Lee P. S. Fully Laser-Patterned Stretchable Microsupercapacitors Integrated with Soft Electronic Circuit Components. NPG Asia Mater. 2018, 10 (10), 959–969. 10.1038/s41427-018-0080-z.

[ref35] Chen H.; Chen S.; Zhang Y.; Ren H.; Hu X.; Bai Y. Sand-Milling Fabrication of Screen-Printable Graphene Composite Inks for High-Performance Planar Micro-Supercapacitors. ACS Appl. Mater. Interfaces 2020, 12 (50), 56319–56329. 10.1021/acsami.0c16976. 33280375

[ref36] Yun J.; Lee H.; Song C.; Jeong Y. R.; Park J. W.; Lee J. H.; Kim D. S.; Keum K.; Kim M. S.; Jin S. W.; Lee Y. H.; Kim J. W.; Zi G.; Ha J. S. A Fractal-Designed Stretchable and Transparent Microsupercapacitor as a Skin-Attachable Energy Storage Device. Chem. Eng. J. 2020, 387, 124076 10.1016/j.cej.2020.124076.

