# Supplementary material for: Design of Porous 3D Interdigitated Current Collectors and Hybrid Microcathodes for Zn-Ion Microcapacitors
Source: ACS Nano. 2025 Mar 25;19(13):13314–24. doi: 10.1021/acsnano.5c00917 (PMC11984303; doi:10.1021/acsnano.5c00917)
Supplement: Supplementary file 1 — nn5c00917_si_001.pdf [file nn5c00917_si_001.pdf]

Support Information for
Design of Porous 3D Interdigitated Current Collectors and Hybrid Micro-Cathodes for Zn-Ion Micro-Capacitors

Yujia Fan,[1] Nibagani Naresh,[1] Yijia Zhu,[1] Mingqing Wang,[1] Buddha Deka Boruah[1,*]

[1]Institute for Materials Discovery, University College London (UCL), London WC1E 7JE, United Kingdom

Corresponding author: Dr. Buddha Deka Boruah

Email: b.boruah@ucl.ac.uk

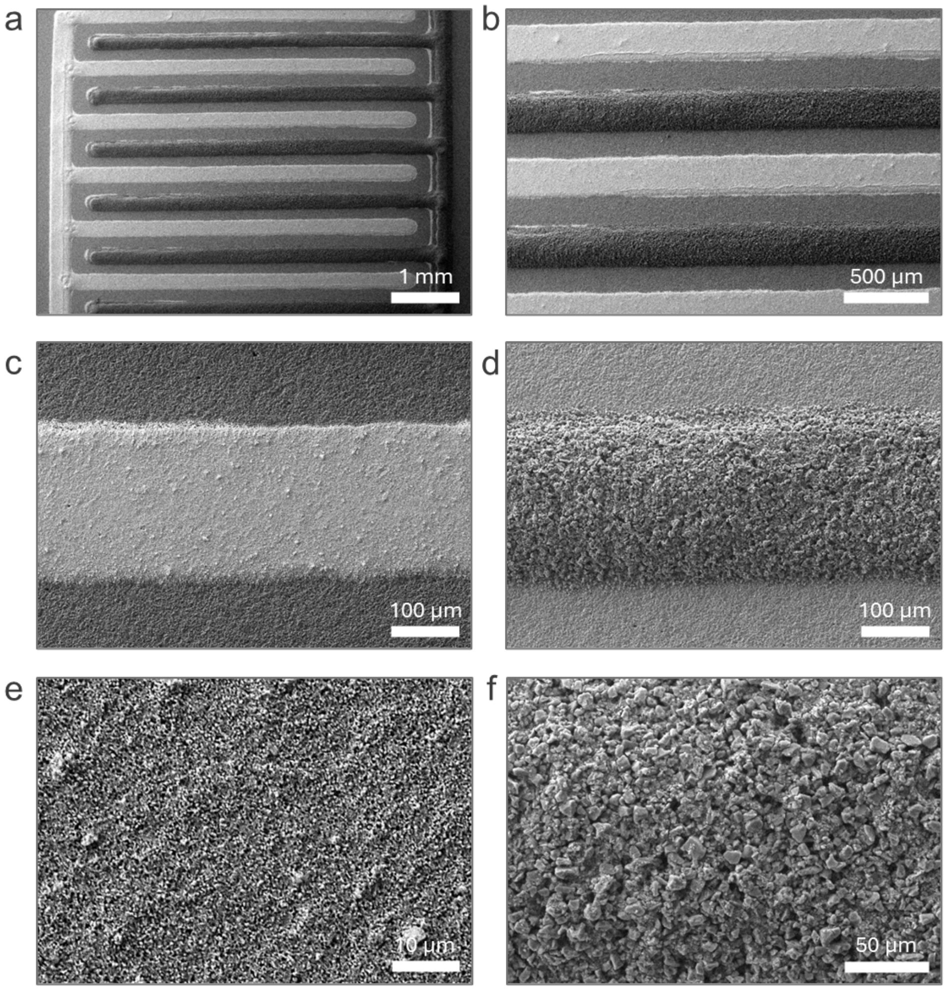

Figure S1. *SEM images of planar Zn//AC ZIMC with different magnification. (a) planar ZIMC ×20. (b) planar ZIMC ×50. (c) Zn anode ×200. (d) AC cathode ×50. (e) Zn anode ×2k. (f) AC cathode ×500*

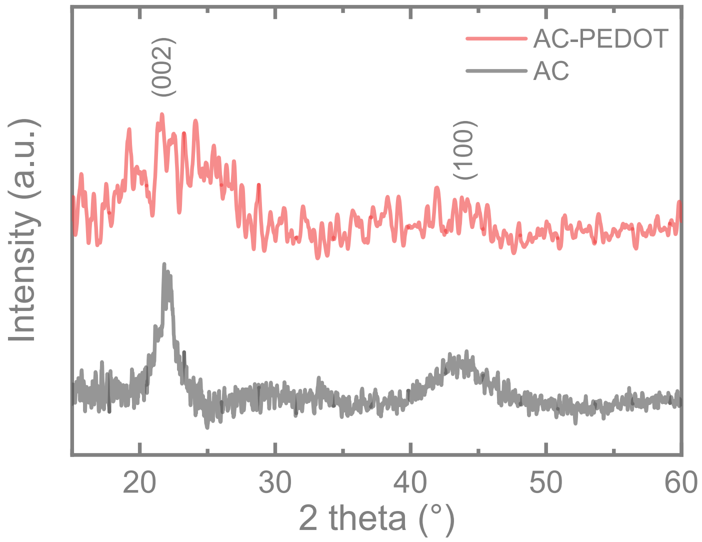

Figure S2. *XRD patterns of AC and AC-PEDOT*

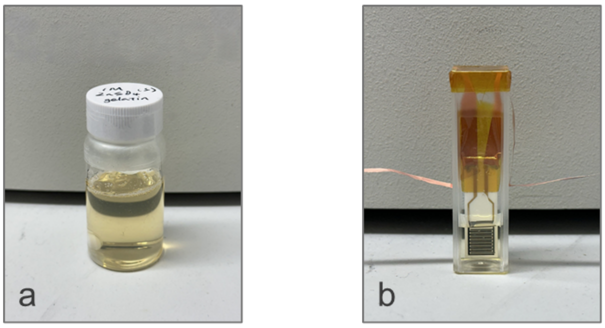

Figure S3. *Digital images of (a) 1M $ZnSO_4$ gelatin electrolyte and (b) ZIMC immersed in the electrolyte.*

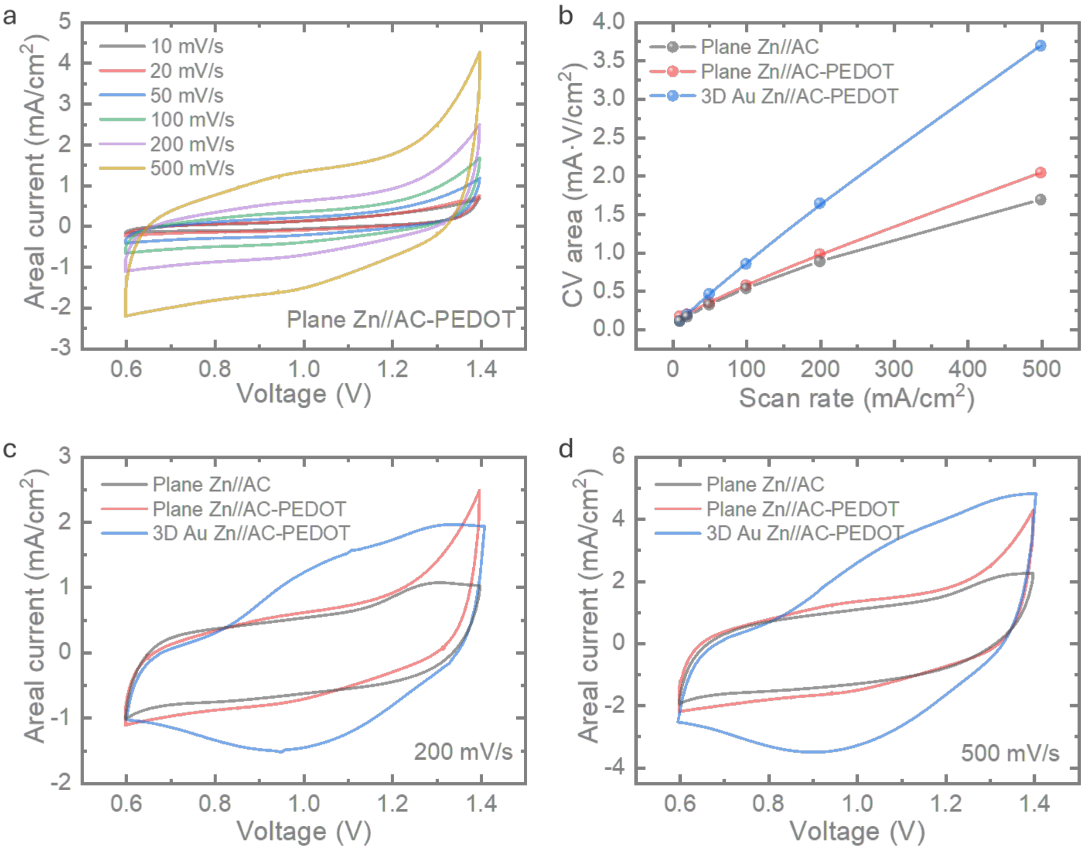

Figure S4*. (a) CV curves of planar Zn//AC-PEDOT recorded at scan rates ranging from 10 to 500 mV/s. (b) CV area as a function of scan rate for planar Zn//AC, planar Zn//AC-PEDOT, and 3D Au Zn//AC-PEDOT. Comparative CV curves of the three ZIMCs at scan rates of (c) 200 mV/s and (d) 500 mV/s.*

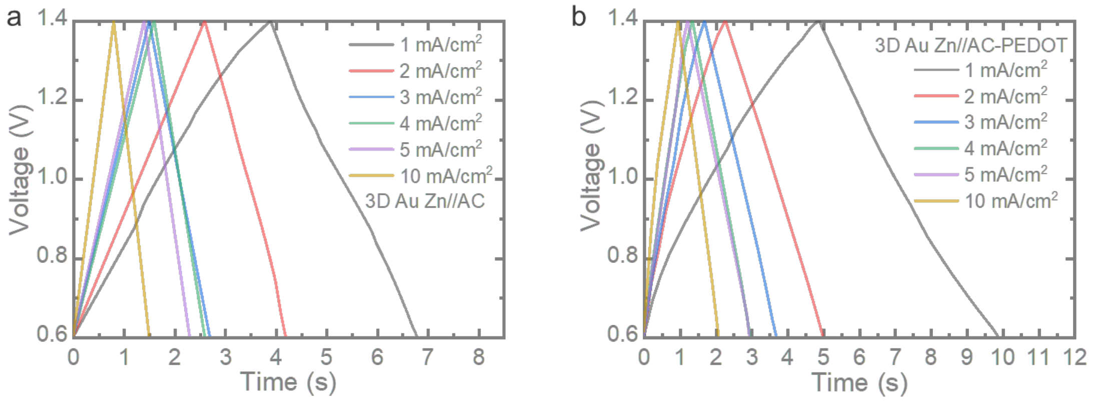

Figure S5. *GCD curves of (a) 3D Au Zn//AC and (b) 3D Au Zn//AC-PEDOT ZIMCs under high areal currents from 1 to 10 mA/cm².*

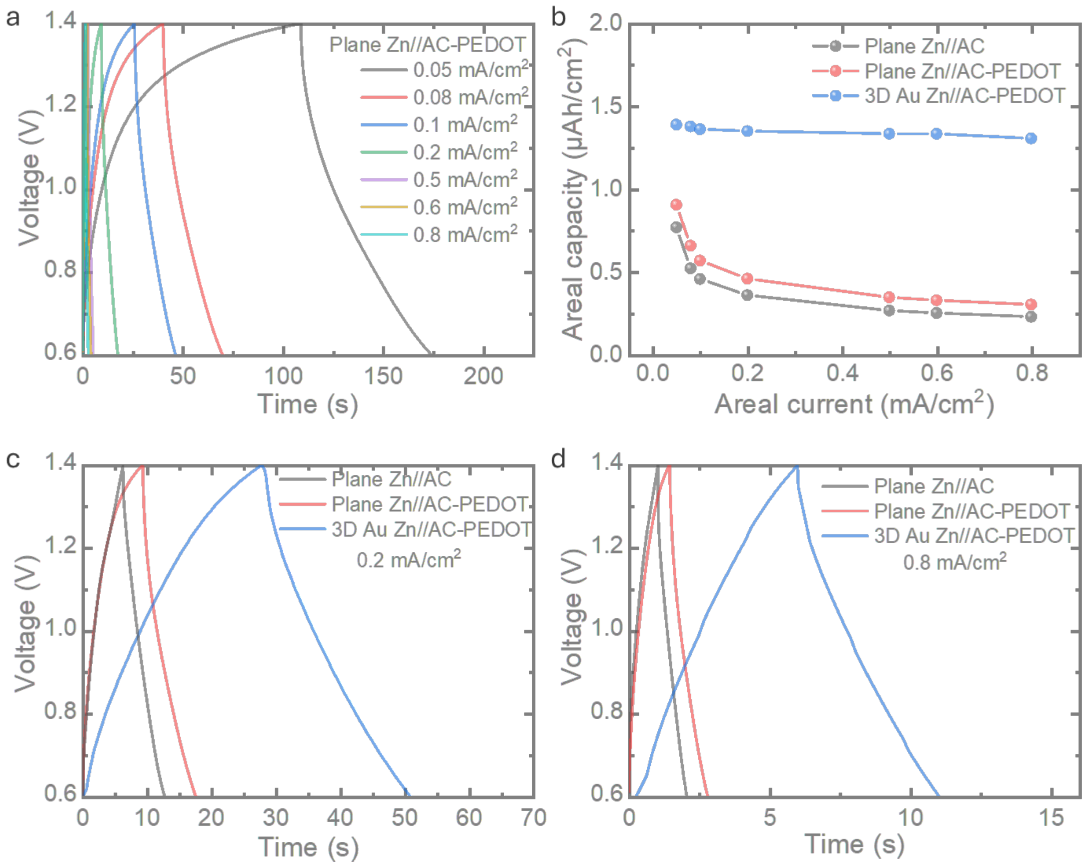

Figure S6. *(a) GCD profiles of planar Zn//AC-PEDOT. (b) Areal capacity as a function of areal current for planar Zn//AC, planar Zn//AC-PEDOT, and 3D Au Zn//AC-PEDOT. Comparative GCD profiles of the three ZIMCs at areal currents of (c) 0.2 mA/cm² and (d) 0.8 mA/cm².*

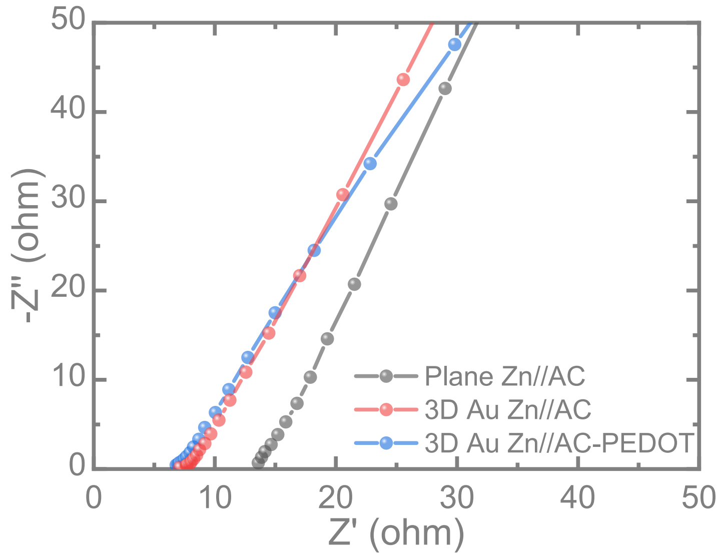

Figure S7. *Nyquist plots of Planar Zn//AC, 3D Au Zn//AC and 3D Au Zn//AC-PEDOT ZIMCs at high frequency region.*

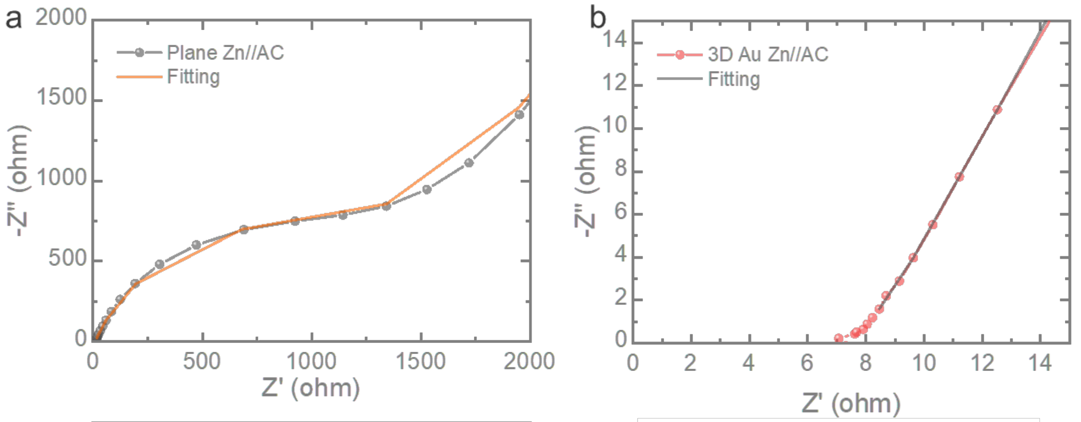

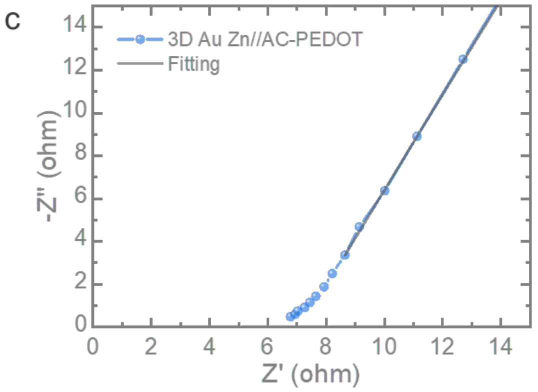

d

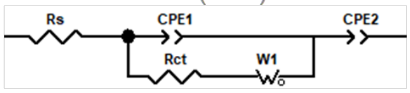

Device	Rs/Ω	Rct
Plane Zn//AC	14.12	1220
3D Au Zn//AC	7.187	8.5
3D Au Zn//AC-PEDOT	6.495	7.581

Figure S8. *Nyquist plots and equivalent circuit analysis of (a) Planar Zn//AC, (b) 3D Au Zn//AC and (c) 3D Au Zn//AC-PEDOT ZIMCs. The lines indicate the fit to the equivalent circuit shown in (d), together with the fitted values for Rs and Rct.*
